# Comparative genomic analysis of light-regulated transcripts in the Solanaceae

**DOI:** 10.1186/1471-2164-10-60

**Published:** 2009-02-03

**Authors:** Mariana Rutitzky, Hernan O Ghiglione, José A Curá, Jorge J Casal, Marcelo J Yanovsky

**Affiliations:** 1 IFEVA, Facultad de Agronomía, Universidad de Buenos Aires and CONICET, Av San Martín 4453, C1417DSE, Buenos Aires, Argentina; 2 Cátedra de Bioquímica, Facultad de Agronomía, Universidad de Buenos Aires, Av. San Martín 4453, C1417DSE, Buenos Aires, Argentina

## Abstract

**Background:**

Plants use different light signals to adjust their growth and development to the prevailing environmental conditions. Studies in the model species *Arabidopsis thaliana *and rice indicate that these adjustments are mediated by large changes in the transcriptome. Here we compared transcriptional responses to light in different species of the Solanaceae to investigate common as well as species-specific changes in gene expression.

**Results:**

cDNA microarrays were used to identify genes regulated by a transition from long days (LD) to short days (SD) in the leaves of potato and tobacco plants, and by phytochrome B (phyB), the photoreceptor that represses tuberization under LD in potato. We also compared transcriptional responses to photoperiod in *Nicotiana tabacum *Maryland Mammoth (MM), which flowers only under SD, with those of *Nicotiana sylvestris*, which flowers only under LD conditions. Finally, we identified genes regulated by red compared to far-red light treatments that promote germination in tomato.

**Conclusion:**

Most of the genes up-regulated in LD were associated with photosynthesis, the synthesis of protective pigments and the maintenance of redox homeostasis, probably contributing to the acclimatization to seasonal changes in irradiance. Some of the photoperiodically regulated genes were the same in potato and tobacco. Others were different but belonged to similar functional categories, suggesting that conserved as well as convergent evolutionary processes are responsible for physiological adjustments to seasonal changes in the Solanaceae. A β-ZIP transcription factor whose expression correlated with the floral transition in *Nicotiana *species with contrasting photoperiodic responses was also regulated by photoperiod and phyB in potato, and is a candidate gene to act as a general regulator of photoperiodic responses. Finally, *GIGANTEA*, a gene that controls flowering time in *Arabidopsis thaliana *and rice, was regulated by photoperiod in the leaves of potato and tobacco and by red compared to far-light treatments that promote germination in tomato seeds, suggesting that a conserved light signaling cascade acts across developmental contexts and species.

## Background

Plant growth and development are shaped by light signals provided by the environment. Seed germination, de-etiolation of aerial tissues, the architecture of the adult plant body and the production of organs involved in sexual or vegetative reproduction are controlled by light signals [1]. The degree of control depends on the species and the process. Studies in *Arabidopsis thaliana *have revealed that large changes in transcriptome accompany the morphological and physiological shifts that occur during the de-etiolation process initiated when dark-grown seedlings are transferred to light [2,3]. Large responses are also observed when young seedlings, briefly grown under white light, are exposed to supplementary far-red light that simulates the presence of neighbour vegetation [4]. The apex of *Arabidopsis thaliana *plants experiences modifications of the transcriptome induced by the exposure of seedlings grown under SD typical of winter to LD that induce flowering during warmer seasons [5]. These large light-induced changes in the transcriptome involve the action of plant photoreceptors at different levels, including the regulation of transcription and proteasome-mediated degradation of transcription factors, chromatin re-modeling and RNA interference (reviewed by [6]).

In addition to the studies conducted in the model eudicot *Arabidopsis thaliana*, others have recently reported global transcriptional responses to light in monocot species. These studies are allowing us to understand species-specific light-regulated processes, such as photoperiodic effects on floret development in wheat [7]. They are also being used for comparative purposes, as shown for the analysis of the transcriptional changes taking place during de-etiolation in rice and *Arabidopsis thaliana *[8].

Functional as well as evolutionary studies can also benefit from the comparison of transcriptional responses across closely-related species [9]. The Solanaceae is an ideal family for comparative analysis of photomorphogenic and photoperiodic responses given that light regulates a variety of process in different species, such as tuberization in potato [10], flowering in tobacco [11] and germination in tomato [12]. Potato cDNA microarrays have already been used to compare global expression profiles in mature leaves of six species of the Solanaceae [13]. Here we compared transcriptional responses to contrasting light environments in the leaves of potato and tobacco plants, as well as in tomato seeds, with the aim of assessing the degree of conservation and divergence in the identity of genes regulated by light across species and developmental contexts.

## Methods

### Plant material and experimental conditions

Plants of *Solanum tuberosum *spp. Andígena, which tuberize only under SD, were grown in growth chambers under non-inductive LD conditions. 55 and 41 days after sowing, half of the plants were transferred to inductive SD conditions for 1 or 15 days, respectively, whilst the rest of the plants were kept as controls under LD. Transgenic potato plants with reduced phyB levels obtained through antisense technology (α-*PHYB*, line 10) [14] were also grown all the time under LD. On the 56th day, leaves and petioles from all the plants were harvested 14 hours after the beginning of the photoperiod (i.e. 2 hours before dusk for plants on LD, and 6 hours after dusk for the plants on SD conditions).

A similar experimental protocol was used with plants of *Nicotiana tabacum *cv Hicks that flower at the same time irrespective of photoperiod and the isogenic line *Nicotiana tabacum *MM, which flowers only under SD (i.e. the plants were grown 55 or 41 days under LD conditions and then transferred for 1 or 15 days to SD, respectively, whilst control plants were grown 56 days under LD).

*Nicotiana sylvestris *plants, which flower only under LD, were grown under non-inductive SD conditions. 55 and 41 days after sowing, half of the plants were transferred to inductive LD conditions for 1 or 15 days respectively. In all cases we harvested only the leaves, the organ in which day-length perception takes place and photoperiodic responses are initiated.

SD in the experiments described above consisted of 8 hours of light/16 hours of darkness, 160 μmol m^-2 ^s^-1^, 22°C. LD conditions were 16 hours of light/8 hours of darkness, 80 μmol m^-2 ^s^-1^, 22°C.

Tomato seeds (La Germinadora, Buenos Aires, Argentina) were imbibed for 16 hours at 20°C under continuous FR (40 μmol m^-2^s^-1^), to standardize initial conditions, eliminating possible maternal effects on the state of phytochromes at the beginning of the experiments [12]. After this imbibition, the seeds were transferred to growth incubators (20°C), where they received hourly pulses (3 minutes each) of R or FR (40 μmoles m^-2^s^-1^), and were harvested in liquid nitrogen 3, 6 and 9 hours after the beginning of the R and FR light pulses. R, compared to FR treatment, was effective in promoting germination (data not shown).

### RNA extraction, microarray processing and data analysis

For gene expression analysis we used the potato cDNA microarray developed by TIGR [15]. Three independent biological samples were analyzed for each treatment. Plant material was grounded under liquid nitrogen and total RNA was extracted with TRIZOL. All steps of microarray processing (cDNA production, cDNA labeling, microarray hybridization, data quantification, data normalization using LOWESS) were carried out by the TIGR Expression Profiling Service [15]. All raw and normalized microarray data is available at: 1) the Solanaceae Gene Expression Database (ID 47 and 52), and 2) The Gene Expression Omnibus (accession number GSE8142).

Genes were considered to be regulated by photoperiod or phyB if the average of the log_2 _(LD/SD) or (WT/α-*PHYB*) ratio was: 1) significantly different from 0 (one sample t-test with a p-value ≤ 0.05 [16] and a q-value ≤ 0.1 [17]), and 2) larger than 1 or smaller than -1 (i.e. there was at least a two fold change in expression). Data from plants exposed to short days for 1 or 15 days were pooled for the analysis of the effect of photoperiod.

To identify genes differentially regulated by photoperiod between species, a t-test (potato vs tobacco) or an ANOVA (among the three *Nicotiana *biotypes evaluated) was performed with the log_2 _(LD/SD) ratios of the species. We considered a gene to be differentially affected by photoperiod if the t-test or the ANOVA gave a p-value ≤ 0.05 and a q-value ≤ 0.1, the gene was considered to be regulated by photoperiod (see criteria above) in at least one of the species and, for the comparison between potato and tobacco, there was a difference of at least 1 unit (two fold) between the log_2 _LD/SD ratios. As mentioned above, data from plants exposed for 1 or 15 days to a change in photoperiodic conditions were pooled for the analysis.

To identify genes regulated by phytochrome in tomato seeds we selected those genes whose expression was statistically affected by pulses of R compared to FR light if: 1) the p-value was ≤ 0.05 in the one sample t-test and 2) there was at least a 1.5 fold change in expression. Data from seeds harvested 3, 6 or 9 hours after the beginning of light treatments were pooled for the analysis of the effect of R compared to FR on gene expression.

### RT-PCR

One μg of DNAseI treated total RNA was used for the RT reaction with ImProm-II Reverse Transcriptase (Promega). Amplification of genomic DNA was undetectable in non-retro-transcribed controls. PCR products were detected in DNA blots using standard methodology in the exponential range of amplification. Primer sequences will be provided upon request.

## Results

### Photoperiodic regulation of gene expression in potato

Potato plants of the subspecies Andígena only tuberize under SD conditions [10]. To investigate the generation of putative signals leading to tuberization, and unrelated transcriptional responses accompanying the acclimatization to a widely different light regime, we analyzed the transcriptome of potato plants transferred from LD to SD. Potato plants were grown in growth chambers under non-inductive LD conditions. After six weeks, half of the plants were transferred to inductive SD for 1 or 15 days, whilst the rest of the plants were kept under LD. RNA was extracted from leaves and petioles harvested 14 hours after the beginning of the photoperiod, and used to analyze gene expression with potato cDNA microarrays developed by TIGR [13].

**Figure 1:**
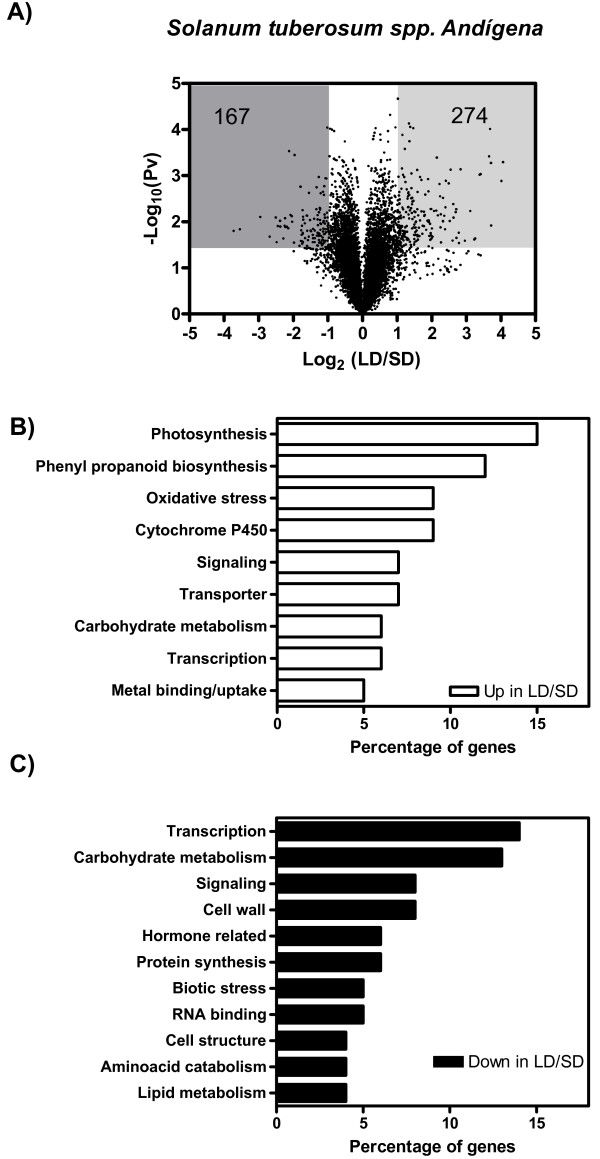
**Effect of photoperiod on gene expression in *Solanum tuberosum *spp. Andígena**. A) Volcano plot of log_2 _transformed expression ratios (LD/SD) plotted against the negative log_10_-transformed p-value from a one-sample t-test. Shaded areas highlight the genes showing a statistically significant difference in gene expression of at least two fold. The numbers indicate the genes present in the shaded area. B) Percentage distribution of functional categories corresponding to genes whose expression decreased in plants transferred from LD to SD conditions. C) Percentage distribution of functional categories corresponding to genes whose expression increased in plants transferred from LD to SD.

Most of the genes that were up or down-regulated when the plants were transferred from LD to SD for 15 days, already showed up or down-regulation after the first day in SD (Additional file 1). Therefore, 1 and 15 days SD were jointly compared to LD. From a total of approximately 10,000 cDNA clones present in the microarray, we found 274 (representing 261 different genes) down-regulated, and 167 (representing 155 different genes) up-regulated in SD compared to LD conditions (Figure 1A).

Many genes down-regulated in SD were associated with the photosynthetic process and phenylpropanoid metabolism (Figure 1B). These findings are in agreement with a previous report indicating that three weeks after *Solanum tuberosum *spp. Andígena plants are transferred from LD to SD, chlorophyll and anthocyanin levels in the leaves of plants grown under SD conditions are 40 and 25% lower, respectively, than those from LD [18]. The expression of several genes involved in the metabolization of reactive oxygen species was also reduced under SD conditions (Figure 1B, Additional file 2). Genes encoding enzymes involved in carbohydrate metabolism were differentially affected by photoperiod (Additional file 2). Within this group, those associated with starch and sucrose biosynthesis were down-regulated, and those involved in starch degradation were up-regulated, in plants grown under SD compared to LD conditions (Additional file 2). Genes associated with cell wall, biotic stress responses, hormone signalling, and aminoacid catabolism were up-regulated in SD compared to LD (Figure 1C, Additional file 2). Finally, the expression of many signaling components and transcription factors increased or decreased in response to changes in photoperiodic conditions (Figure 1B and 1C, Additional file 2). Some of these changes may be associated with the regulation of developmental processes such as tuberization (see below), whilst others may control more general metabolic and physiological adaptations to photoperiod. For instance, the expression of two genes encoding AMP-activated protein kinases was up-regulated under SD (Additional file 2). AMP-activated kinases are known to turn-off energy dependent processes and mobilize energy reserves under low energy conditions [19]. Thus, its induction under SD conditions may contribute to optimize energy consumption during the autumn.

### Analysis of phytochrome B-regulated gene expression in potato

**Figure 2:**
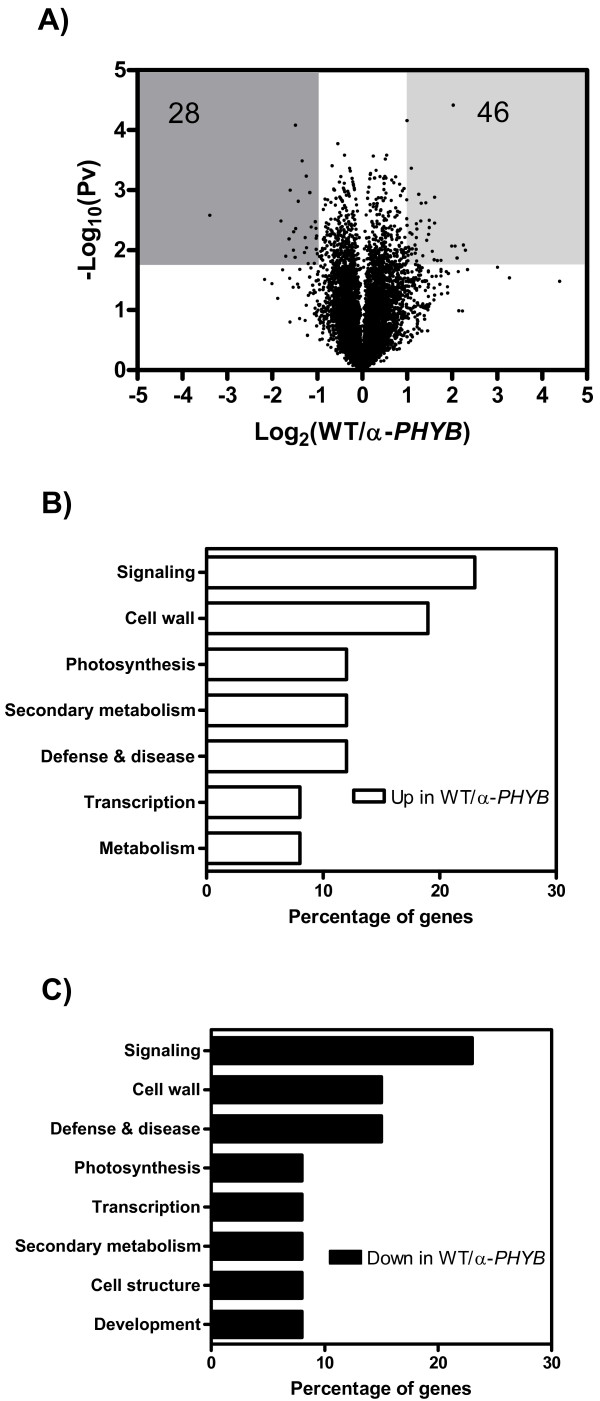
**Effect of phyB on gene expression in *Solanum tuberosum *spp. Andígena**. A) Volcano plot of log_2 _transformed expression ratios (WT/α-*PHYB*) plotted against the negative log_10_-transformed p-value from a one-sample t-test. Shaded areas highlight the genes showing a statistically significant difference in gene expression of at least two fold. The numbers indicate the genes present in the shaded area. B) Percentage distribution of functional categories corresponding to genes whose expression was higher in WT compared to α-*PHYB *plants. C) Percentage distribution of functional categories corresponding to genes whose expression was higher in α-*PHYB *compared to WT plants.

PhyB is the main photoperiodic photoreceptor regulating tuberization in potato [14]. Indeed, potato plants with reduced phyB levels obtained through antisense technology tuberize as well under LD as under SD, whilst wild-type plants only tuberize under SD [14]. To elucidate the genomic role of phyB in the perception of LD as well as in the control of genes unaffected by day-length, we compared the transcriptome of wild-type and antisense *PHYB *plants grown under LD conditions (Figure 2). We found 46 cDNA clones (representing 45 different genes) up-regulated and 28 cDNA clones (representing 27 different genes) down-regulated in WT plants compared to α-*PHYB *plants (Figure 2A). In contrast to what was observed for the group of genes regulated by photoperiod, no clear differential enrichment in functional categories was found for genes up-regulated compared to those down-regulated by phyB (Figure 2B). Nonetheless, some of the individual changes in gene expression observed fit well with physiological alterations reported for plants with reduced phyB levels (Additional file 3). For example, the expression of a senescence associated gene, which encodes an acyl-hydrolase that facilitates the process of membrane breakdown [20], was up-regulated in plants with reduced phyB levels and this is in agreement with the observation that leaf senescence is enhanced by low levels of active phytochromes in several species [21,22]. In addition, the expression of a gene encoding a pathogen related gene was down-regulated in α-*PHYB *compared to WT plants. Indeed, *PR *gene expression is affected in *Arabidopsis thaliana *mutant plants lacking phyA and phyB, and these phytochrome mutants have reduced defense responses against pathogen attacks [23-25].

**Figure 3:**
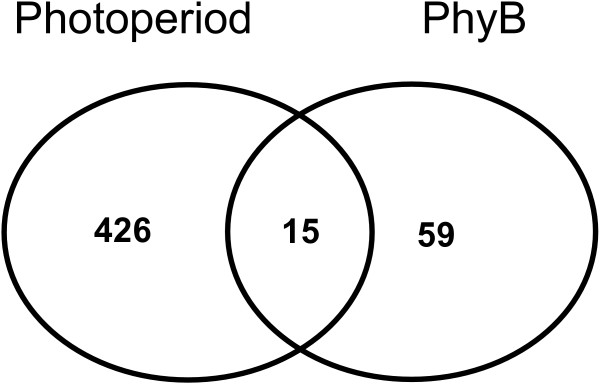
**Comparative analysis of the effect of photoperiod and phyB on gene expression in potato**. The genes present in the graph are those whose expression was regulated by photoperiod and/or phyB, according to the criteria used for Figures 1 and 2.

Since phyB mediates the photoperiodic control of tuberization, we expected a strong overlap between genes regulated by photoperiod and phyB. Strikingly, from a total of 441 cDNA clones significantly regulated by photoperiod, only 15 were significantly affected by phyB levels (Figure 3, Additional file 4). These results indicate that although phyB is the main photoperiodic photoreceptor regulating the tuberization process, photoreceptors other than phyB play a significant role in the photoperiodic regulation of gene expression, probably associated with acclimatization responses to reduced light input rather than to the control of tuberization.

**Figure 4:**
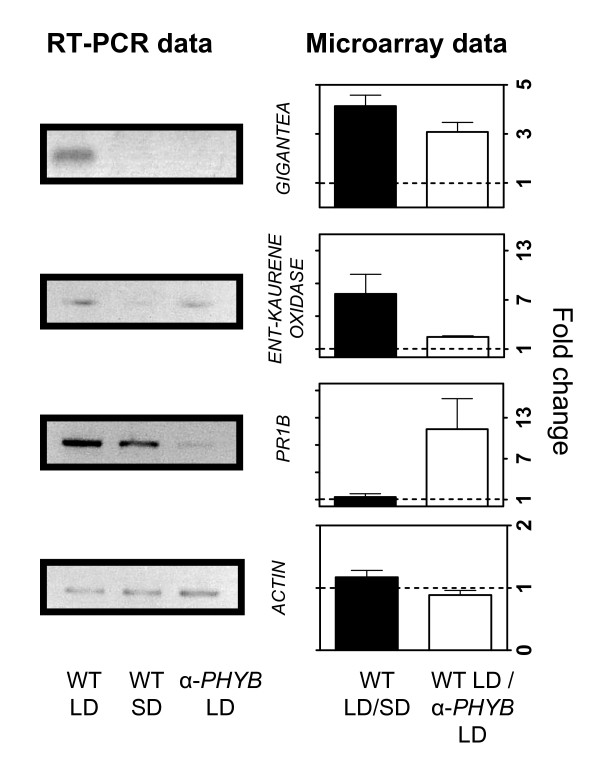
**RT-PCR and microarray expression data of selected genes showing different patterns of phyB and photoperiodic regulation of expression**. The expression of *GIGANTEA*, *ENT-KAURENE OXIDASE*, *PR1B *and *ACTIN *was analyzed by RT-PCR in wild-type plants grown under LD or SD conditions, as well as in α-*PHYB *grown under LD. RT-PCR data is shown on the left panels and the corresponding microarray data for each gene is displayed on the right panels.

Among the 15 genes identified as simultaneously regulated by photoperiod and phyB, two are candidates to mediate the photoperiodic control of tuberization. These are an homologue of *GIGANTEA *and a gene encoding the enzyme ent-kaurenoic acid oxidase. *GIGANTEA *promotes flowering under LD in *Arabidopsis thaliana *(a LD plant) and represses flowering under LD in rice (a SD plant) [26]. The expression of a homologue of *GIGANTEA *in potato was higher under LD compared to SD, and higher in wild type plants compared to transgenic plants with reduced phyB levels when both genotypes are grown under LD (Figure 4). This observation suggests that *GIGANTEA *could be mediating the repression of tuberization by LD in potato.

Gibberellins accumulate under LD in potato and inhibit the tuberization process [10]. Here we found that a gene encoding the enzyme ent-kaurenoic acid oxidase, which controls an early step in the gibberellin biosynthetic pathway, was up-regulated under LD compared to SD conditions and in wild-type plants compared to plants with reduced phyB levels (Figure 4). Thus, ent-kaurenoic acid oxidase may be one of the biochemical steps of the GA metabolic pathway through which photoperiod regulates gibberellin biosynthesis and tuberization in potato.

To validate the microarray data we analyzed the expression of genes differentially regulated by photoperiod and/or phyB through RT-PCR. Indeed, we confirmed that the expression of *GIGANTEA *and *ENT-KAURENOIC ACID OXIDASE *genes was higher in plants grown under LD compared to SD. The expression of *GIGANTEA *and, to a lesser extent, the expression of an *ENT-KAURENOIC ACID OXIDASE *gene was also higher in wild type plants than in plants with reduced phyB levels. Finally, the expression of a *PR1b *gene was higher in wild-type plants than in α-*PHYB *plants, but was not affected by photoperiod, as observed in the microarray data (Figure 4).

### Transcriptomic changes in response to photoperiod in tobacco

A change from LD to SD, in addition to promoting tuberization in potato, also induces flowering in *Nicotiana tabacum *MM [11]. In order to evaluate the degree of conservation and divergence in the transcriptomic responses to photoperiod in closely related species, we evaluated the changes in gene expression that took place when plants of *Nicotiana tabacum *MM grown under LD were transferred to SD conditions, using the same experimental protocol described for potato plants. Because microarrays specific for *Nicotiana tabacum *are not available, gene expression results were obtained by hybridizing tobacco samples to *Solanum tuberosum *arrays. Recent experiments have indicated that cross-species hybridization give results that closely match those obtained with species-specific probes when fold changes in expression between control and treatments are analyzed within a given species [27].

**Figure 5:**
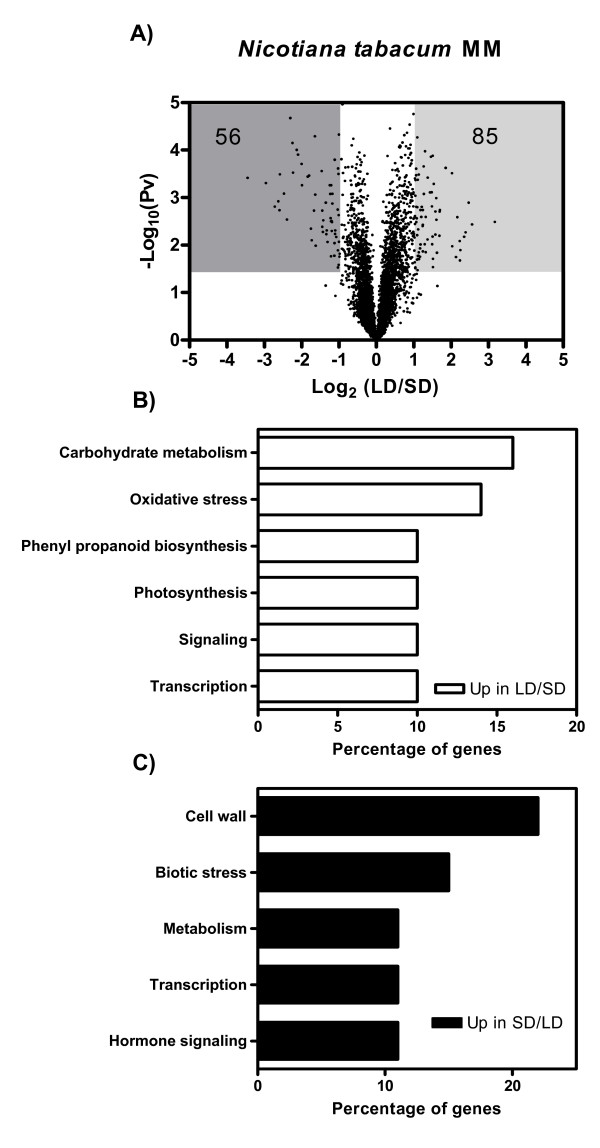
**Effect of photoperiod on gene expression in *Nicotiana tabacum *MM**. A) Volcano plot of log_2 _transformed expression ratios (LD/SD) plotted against the negative log_10_-transformed p-value from a one-sample t-test. Shaded areas highlight the genes showing a statistically significant difference in gene expression of at least two fold. The numbers indicate the genes present in the shaded area. B) Percentage distribution of functional categories corresponding to genes whose expression decreased in plants transferred from LD to SD conditions. C) Percentage distribution of functional categories corresponding to genes whose expression increased in plants transferred from LD to SD.

As observed in potato plants, most of the genes whose expression was higher under LD compared to SD in *Nicotiana tabacum *MM were associated with photosynthesis, phenylpropanoid metabolism, carbohydrate metabolism (starch and sucrose biosynthesis) and oxidative stress (Figure 5; Additional file 5). The majority of the genes up-regulated under SD compared to LD included those encoding cell wall modifying enzymes as well as genes associated with biotic stress responses (Figure 5, Additional file 5).

The expression of several genes encoding transcription factors and signalling molecules was affected when the plants were changed from LD to SD, and may mediate some of the developmental as well as physiological responses to photoperiod. For example, the expression of *GIGANTEA *was up-regulated under LD (Additional file 5), and this change is likely to contribute to the photoperiodic regulation of flowering [28]. In addition, a gene whose expression was down-regulated more than 4 fold after transferring the plants from non inductive LD to inductive SD encodes a CCAAT transcription factor (Additional file 5). The gene with the highest degree of similarity in *Arabidopsis thaliana *is *At5g12840*, which encodes a HAP2 protein that delays flowering when over-expressed in transgenic plants [29]. Thus, down-regulation of the HAP2 homologue in *Nicotiana tabacum *MM could be involved in the promotion of flowering by SD in these plants. Finally the expression of a gene encoding an EIN3 homologue is up-regulated under SD (Additional file 5). EIN3 homologues have been recently shown to promote the expression of several pathogen related proteins in tobacco [30]. Thus the enhanced expression of an EIN3 homologue may be causing the overrepresentation of biotic stress related genes in tobacco plants transferred to SD.

**Figure 6:**
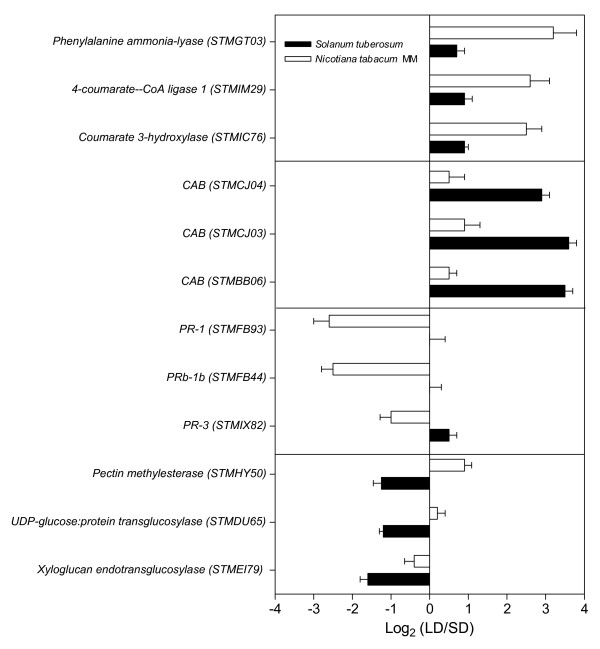
**Comparative analysis of the effect of photoperiod on gene expression between *Nicotiana tabacum *MM and *Solanum tuberosum *spp. Andígena**. The genes displayed belong to functional categories containing at least three genes showing differential regulation by photoperiod between potato and tobacco. For each functional category, the three genes with the highest change in expression in response to photoperiod are presented.

In spite of the significant overlapping in functional categories regulated by photoperiod in tobacco and potato, only 35% of the genes regulated by photoperiod in tobacco were significantly regulated by photoperiod in potato. In particular, many of the phenylpropanoid and biotic stress-associated genes regulated by photoperiod in tobacco were not affected in potato (or were affected to a significantly lesser extent) and the opposite occurs for many genes associated with the photosynthetic process and cell-wall modifying enzymes (Figure 6, Additional file 6).

### Genes with opposite photoperiodic regulation in Nicotiana biotypes with contrasting flowering response types

**Figure 7:**
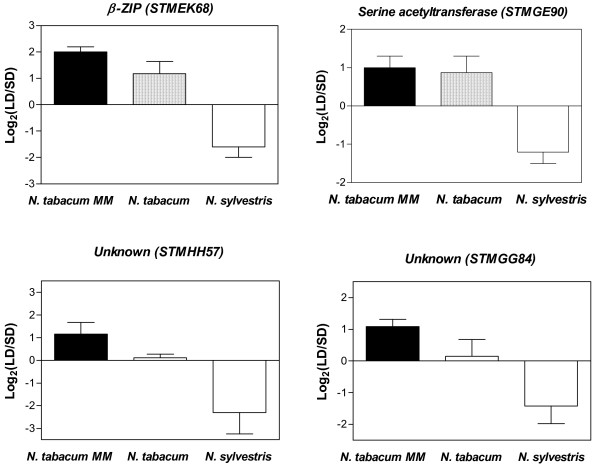
**Genes showing opposite responses to photoperiod in *Nicotiana *biotypes with contrasting photoperiodic regulation of flowering time**. Log_2 _transformed expression ratios (LD/SD) of selected genes in *Nicotiana tabacum *MM (a short-day plant), *Nicotiana tabacum *(a day-neutral plant) and *Nicotiana sylvestris *(a long-day plant).

Many *Nicotiana *species and cultivars exhibit different floral responses to photoperiod. *Nicotiana tabacum *cv Hicks flower at the same time under LD and SD conditions, *Nicotiana tabacum *cv Hicks MM flower only under SD, whilst *Nicotiana sylvestris *flower only under LD [31]. Grafting experiments show that the substances that promote or inhibit flowering can be transferred among plants irrespective of their response type, indicating that the inhibitory or stimulatory substances are similar or identical [31]. To identify genes whose expression could be associated with the regulation of the floral transition, we compared the transcriptional changes that took place in the leaves of the *Nicotiana *biotypes described above, when the plants were transferred from non-inductive to inductive conditions. Using ANOVA we found 52 genes for which the photoperiodic regulation of expression was significantly different among biotypes and showed at least a two fold change in expression in one of them (Additional file 7). The majority of these genes showed at least a two fold change in expression in either *Nicotiana tabacum *MM or *Nicotiana sylvestris*, but not in both species simultaneously, making uncertain whether they played a role in the contrasting photoperiodic regulation of flowering time among species. In contrast, four genes were up-regulated more than two fold under LD in *Nicotiana tabacum *MM (a SD plant) and under SD in *Nicotiana sylvestris *(a LD plant), suggesting that they might act as repressors of the floral transition in these plants (Figure 7). These genes encode a β-ZIP transcription factor, a serine acetyltransferase, and two proteins of unknown function. The expression of the β-ZIP transcription factor, as well as the expression of one unknown gene (cDNA STMGG84), was also regulated by photoperiod and phyB in potato (Additional files 2 and 3), suggesting they may regulate the tuberization process.

### Light induced changes in gene expression in tomato seeds

**Figure 8:**
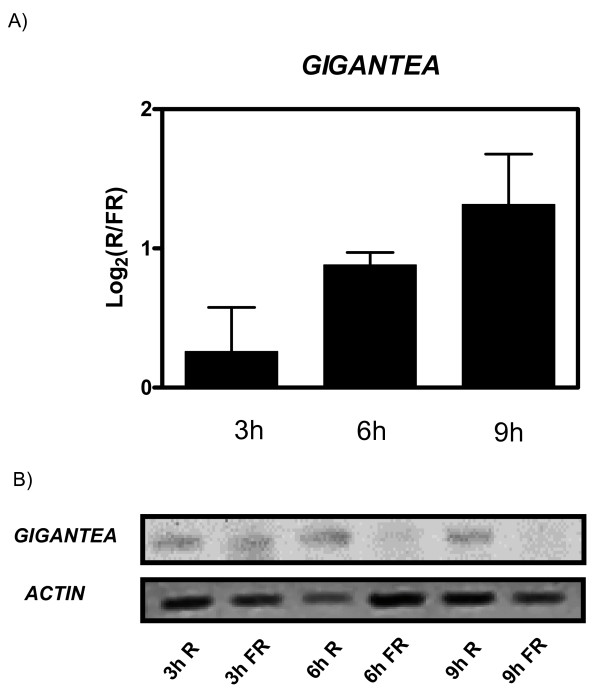
**Effect of R compared to FR on the expression of *GIGANTEA *in tomato seeds**. A) microarray and B) RT-PCR expression data for *GIGANTEA *in tomato seeds exposed for 3, 6, or 9 hours to contrasting R and FR treatments.

To expand the range of light-regulated developmental processes and species investigated we analyzed transcriptional changes associated with the promotion of tomato seed germination by red compared to far-red light perceived by the phytochrome photoreceptors [12]. For this we exposed tomato seeds to short pulses of red or far-red light for 3, 6 or 9 hours, extracted total RNA from whole seeds, and used the RNA to analyze gene expression with potato cDNA microarrays. Using this approach we could identify only 4 genes regulated more than 1.5 fold by light (Additional file 8). The reduced impact of light on gene expression in tomato seeds, compared to its larger effect in potato and tobacco leaves, could be due to the fact that regulation of germination in tomato most likely involves molecular changes taking place specifically in a few cells in the micropylar region of the endosperm [32], but we used total RNA extracted from whole seeds for our analysis. In addition, it is likely that some of the genes involved in the regulation of seed germination are specifically expressed in seeds, and are not represented in the potato cDNA microarray. In spite of the limitations just described, some of the genes identified are likely to have a role in the promotion of germination by red light. One of the genes up-regulated in red compared to far-red light encodes a glucan endo-1,3-beta-glucosidase, and increases in the protein levels of a similar protein have already been reported to occur in tobacco and *Arabidopsis thaliana *seeds during germination [33]. The increase in expression of this glucan endo-1,3-beta-glucosidase may play a role hydrolizing the cell walls of endosperm cells, thus facilitating radicle emergence. Interestingly, the other gene up-regulated by red compared to far-red light in tomato seeds encodes a homologue of *GIGANTEA*, which we found to be regulated by photoperiod and phyB in potato and by photoperiod in the leaves of tobacco plants (Figure 4). Regulation of *GIGANTEA *expression by red compared to far-red light in tomato seeds was confirmed by RT-PCR (Figure 8), indicating that its control by light is indeed conserved across species and developmental contexts.

## Discussion

DNA microarrays have been used recently to analyze transcriptional changes associated with photomorphogenic processes in plants, with the majority of them conducted in *Arabidopsis thaliana*. Here we expanded the application of functional genomic approaches to photomorphogenic studies, by using potato cDNA microarrays developed by TIGR to characterize transcriptional changes taking place in different species of the Solanaceae, in response to different light treatments, and across several developmental contexts.

### Acclimatization to seasonal changes in potato and tobacco

Whilst significant progress has been made in recent years towards understanding the molecular mechanism of the photoperiodic regulation of flowering time [34], little is known about more general biochemical and physiological acclimatization responses to changes in photoperiod that allow plants to cope with seasonal variations in light intensity, temperature and humidity. Furthermore, although it is well established that the perception of photoperiod takes place in the leaves [35], no single study has analyzed so far the effect of photoperiod on gene expression levels in the leaves of any plant species.

In this study we have identified hundreds of genes whose expression differed between the leaves of plants grown under LD and SD conditions, when compared 14 hours after the beginning of the photoperiod (i.e. 2 hours before lights off in LD and 6 hours after lights off in SD). These differences in expression could result from direct effects of light on gene expression, and/or from interactions between light and the circadian clock (e.g. from effects of light on the amplitude and/or phase of circadian rhythms in gene expression). An evaluation of gene expression data spanning a complete day would be required to investigate the above options in more detail.

Many genes associated with the photosythetic apparatus and the synthesis of protective pigments were down-regulated under SD compared to LD conditions. Genes associated with redox metabolism were also down-regulated in SD compared to LD. All the above indicates that a major part of the transcriptional changes taking place during the transition from LD to SD is associated with a reduction in the synthesis of proteins that cooperate to convert solar into chemical energy, as well as in pigments and redox regulating enzymes needed to protect plants from the damaging effects of excess of radiant energy that plants receive under LD. These results are in agreement with a recent study conducted in *Arabidopsis thaliana*, showing that the endogenous system that measures day-length interacts strongly with redox regulatory mechanism [36]. The later study shows that plants grown under LD constitutively display systems for the prevention of oxidative damage and show no further responses to increases in radiant energy. On the other hand, plants grown under SD invest less resources in preventing oxidative damages when grown under low to moderate irradiances, but show strong increases in antioxidant mechanisms when exposed to high levels of radiant energy [36].

Another interesting observation from our microarray dataset was that genes associated with aminoacid catabolism were up-regulated under SD compared to LD in potato plants. Up-regulation of this gene class has already been reported to occur in *Arabidopsis thaliana *in response to extended darkness and sugar starvation[37]. Our results suggest that an increase in aminoacid catabolism genes is a general acclimatization response that may help plants adjust carbon and energy metabolism in response to sugar starvation conditions associated with the shortening of the day. Candidate genes to mediate the regulation of the above changes are those encoding subunits of AMP-activated kinases, whose expression also increased under SD. Interestingly, it has been reported recently that a *Physcomitrella patens *mutant lacking two AMP-activated kinases only grows well under continuous light but is unable to grow under light-dark cycles [38]. Therefore, changes in transcript levels of genes encoding AMP-activated kinases may play a significant signaling role adjusting the carbon and energy metabolism of plants to the low energy condition resulting from short photoperiods.

### Evolutionary origins of the photoperiodic regulation of the transcriptome in the Solanaceae

The comparison of transcriptomic responses to changes in photoperiod in potato and tobacco offered an interesting opportunity to explore the evolutionary origins of light regulated responses in the Solanaceae. It is generally believed that similar phenotypes in closely related species are the consequence of conserved evolutionary processes. Indeed, several genes associated with redox homeostasis, sugar metabolism and the photosynthetic process were similarly regulated by photoperiod in potato and tobacco, suggesting an ancient evolutionary origin for the regulation of those metabolic and physiological processes. However, a common adaptive response to a similar environmental challenge can also arise through convergent evolutionary processes involving different molecular mechanisms. One of the most common responses of plants to the excess of light to which they are exposed during the LD of the summer is the accumulation of protective pigments derived from the phenylpropanoid biosynthetic pathway. Here we show that several of the genes associated with the phenylpropanoid biosynthetic pathway that were regulated by photoperiod differed between tobacco and potato plants. For example, the expression of a gene encoding a phenylalanine ammonia-lyase enzyme was strongly regulated by photoperiod in tobacco but not in potato. The converse occurred for a gene encoding a flavonoid 3'-hydroxylase enzyme (Additional file 9). These observations strongly suggest that the molecular mechanisms leading to the accumulation of pigments that can protect plants from the excess of radiant energy during the summer might be the result, at least in part, of independent but convergent evolutionary processes in potato and tobacco. A similar phenomenon has been described in mice, where the evolution of the pigmentation phenotype in two closely related species appears to have a different genetic origin [39,40].

### GIGANTEA, a signaling component associated with multiple phytochrome-regulated developmental processes in the Solanaceae

The photoperiodic regulation of tuberization in potato is a well studied process at the physiological level, but the molecular mechanisms underlying it are only beginning to be understood [10,41]. PhyB has been shown to inhibit tuberization under LD, promoting the synthesis of an inhibitor of the tuberization process, although the molecular nature of this inhibitor remains elusive [42]. We found that, among the 416 different genes (represented in 441 cDNA clones) whose expression was regulated by photoperiod in potato leaves, 15 genes were also regulated by phyB. Among these we found *GIGANTEA*, a gene that promotes flowering in *Arabidopsis thaliana*. *GIGANTEA *positively regulates the expression of *CONSTANS*, a transcriptional regulator that promotes floral induction when its protein accumulates above a threshold level. Indeed, over-expression of the *Arabidopsis thaliana CONSTANS *gene in potato plants delays tuberization [10]. Since tuberization is induced under SD, the up-regulation of *GIGANTEA *under LD compared to SD is likely to repress the tuberization process, presumably through the regulation of *CONSTANS *expression.

Interestingly, we also found *GIGANTEA *as one of the genes whose expression was up-regulated by red light in tomato seeds. In agreement with this observation, we have observed that, at least in *Arabidopsis thaliana*, *GIGANTEA *mediates the promotion of germination triggered by light pulses perceived by phytochrome A[43]. The results presented here suggest that, at least in tomato, *GIGANTEA *may mediate the red-light promotion of germination that is expected to be controlled by phyB. *GIGANTEA *has already been shown to play a positive role in phyB mediated de-etiolation in *Arabidospsis thaliana *[44]. Furthermore, our results show that the expression of *GIGANTEA *is regulated by phyB in the leaves of potato plants. Thus, *GIGANTEA *is likely to play a key role mediating different phytochrome regulated processes in different species.

### Identifying candidate photoperiodic regulatory genes through comparative functional genomics

Changes in photoperiod regulate flowering time in many species [35]. Most of the genes that mediate the photoperiodic regulation of flowering have been identified during the last decade through forward genetic approaches using *Arabidopsis thaliana *and rice as model systems. The identification of photoperiodic regulated genes can also be a useful approach to find new flowering time genes. However, genes whose expression is regulated by photoperiod may not only act regulating developmental transitions, but also other unrelated physiologic and metabolic processes. One way of overcoming the above problem is comparing gene expression in plants with contrasting photoperiodic responses and identifying those genes whose expression correlates with the final response (promotion or repression of floral transition), rather than with the actual photoperiodic condition under which the plants are growing. An example of such gene is *FT*, whose mRNA increases in the leaves of the long-day *Arabidopsis thaliana *plants under LD conditions, whilst the mRNA of an *FT *orthologue increases under SD in the leaves of the short-day rice plants [26]. Another example is the *FLOWERING PROMOTING FACTOR 1 *gene from tobacco, whose overexpression accelerates flowering in *Nicotiana *species with contrasting photoperiodic response types [45]. Furthermore, the expression of this gene increases in the apices of the SD plant *Nicotiana tabacum *MM during growth under SD, as well as in the apices of the LD plant *Nicotiana sylvestris*, when the later is grown under LD [45].

Here we compared global changes in gene expression in the SD plant *Nicotiana tabacum *MM and in the LD plant *Nicotiana sylvestris*, when the plants were moved from non-inductive to inductive conditions for the floral transition. This approach allowed us to identify four genes whose expression was anti-correlated with the floral induction process. One of the genes identified encodes a β-ZIP transcription factor. In *Nicotiana tabacum *MM, the expression of this gene was higher under LD compared to SD, and in *Nicotiana sylvestris *its expression was higher under SD compared to LD. Thus, this gene is likely to encode a repressor of the floral transition. In principle, a transcription factor repressing the floral transition could operate promoting the expression of a floral inhibitor or repressing that of a floral promoter. Transmission of flower-promoting materials through grafting experiments have been demonstrated for both *Nicotiana tabacum *MM and *Nicotiana sylvestris*, whilst transmition of flower-inhibiting substances have only been observed for *Nicotiana sylvestris *[31]. The latest observation suggests that the β-ZIP identified here might repress the expression of a floral-promoting factor in both *Nicotiana *species.

Physiological as well as molecular evidence indicates that the factors mediating the photoperiodic regulation of flowering and tuberization may be similar or identical [10,46]. If this were the case, some of the genes that we identified as candidates to regulate flowering time in *Nicotiana *species may also be candidates to control tuberization in potato. Interestingly, we found that the expression of the β-ZIP transcription factor decreased in the leaves of potato plants that were transferred from LD to SD and was also higher in WT plants compared to transgenic plants with reduced phyB levels. Thus this gene is a good candidate to act not only as a flowering time regulator but as general regulator of photoperiodic responses in the Solanaceae. Reverse genetic approaches are under way to evaluate the role of this β-ZIP transcription factor in the photoperiodic control of plant development.

## Conclusion

The use of cDNA microarrays allowed us to identify hundreds of genes that were regulated by light in different species of the Solanaceae. Many genes were regulated by photoperiod in potato, and a few of those were also regulated by phyB (the main photoperiodic photoreceptor controlling tuberization), making them good candidates to act as developmental regulators. The comparison of photoperiodically regulated genes between potato and tobacco revealed conserved, but also species-specific responses, showing that adaptations to changes in the light environment have evolved multiple times and represent a mixture of ancient as well as recent evolutionary processes. Finally, we found a few genes regulated by light across developmental contexts and species. Some of these are homologues of genes previously found to play critical roles in light signaling in *Arabidopsis thaliana *and rice, whilst others are proposed to play regulatory roles in light signaling for the first time in this work. Thus, the use of a comparative functional genomic approach appears to be a useful tool to enhance our understanding of the evolutionary mechanisms underlying adaptation of plants to changes in the light environment, as well as to identify signaling regulators.

## Competing interests

The authors declare that they have no competing interests.

## Authors' contributions

MJY and JJC developed the experimental design. MR and HOG conducted the experiments. MR, HOG, JAC, JJC and MJY analyzed the data. MR, JJC and MJY drafted the manuscript. All authors read and approved the final manuscript.

## Supplementary Material

Additional file 1 **Correlative analysis of the effect of 1 or 15 SD on gene expression in potato plants.** XY graph comparing the expression ratios (log_2_LD/SD) of potato plants transferred from LD to SD conditions for 1 (x-axis) or 15 (y-axis) days. Click here for file

Additional file 2 **Effect of photoperiod on gene expression in potato.** Table of genes whose expression was considered to be significantly affected by photoperiod in potato plants. Click here for file

Additional file 3 **Effect of phyB on gene expression in potato.** Table of genes whose expression was considered to be significantly affected by phyB in potato plants. Click here for file

Additional file 4 **Overlapping effects of photoperiod and phyB on gene expression in potato.** Table of genes whose expression was considered to be significantly affected by photoperiod and phyB in potato plants. Click here for file

Additional file 5 **Effect of photoperiod on gene expression in *Nicotiana tabacum *MM.** Table of genes whose expression was considered to be significantly affected by photoperiod in *Nicotiana tabacum *MM. Click here for file

Additional file 6 **Contrasting regulation of gene expression by photoperiod in potato and tobacco.** Table of genes whose expression was considered to be differentially affected by photoperiod between potato and tobacco plants. Click here for file

Additional file 7 **Genes differentially regulated by photoperiod among *Nicotiana *biotypes.** Table of genes whose expression was considered to be differentially affected by photoperiod among *Nicotiana *biotypes with contrasting photoperiodic regulation of flowering time. Click here for file

Additional file 8 **Effect of phytochrome on gene expression in tomato seeds.** Table of genes whose expression was considered to be significantly affected by red compared to far-red light in tomato seeds. Click here for file

Additional file 9 **Contrasting response to photoperiod in potato and *Nicotiana tabacum *MM.** Expression data corresponding to genes of the phenylpropanoid biosynthetic pathway that showed differential responses to photoperiod between potato and tobacco. Click here for file
