# Supplementary figures and images for: Comparative genomic analysis of light-regulated transcripts in the Solanaceae
Source: BMC Genomics. 2009 Feb 3;10:60. doi: 10.1186/1471-2164-10-60 (PMC2644711; doi:10.1186/1471-2164-10-60)

## Slide 1
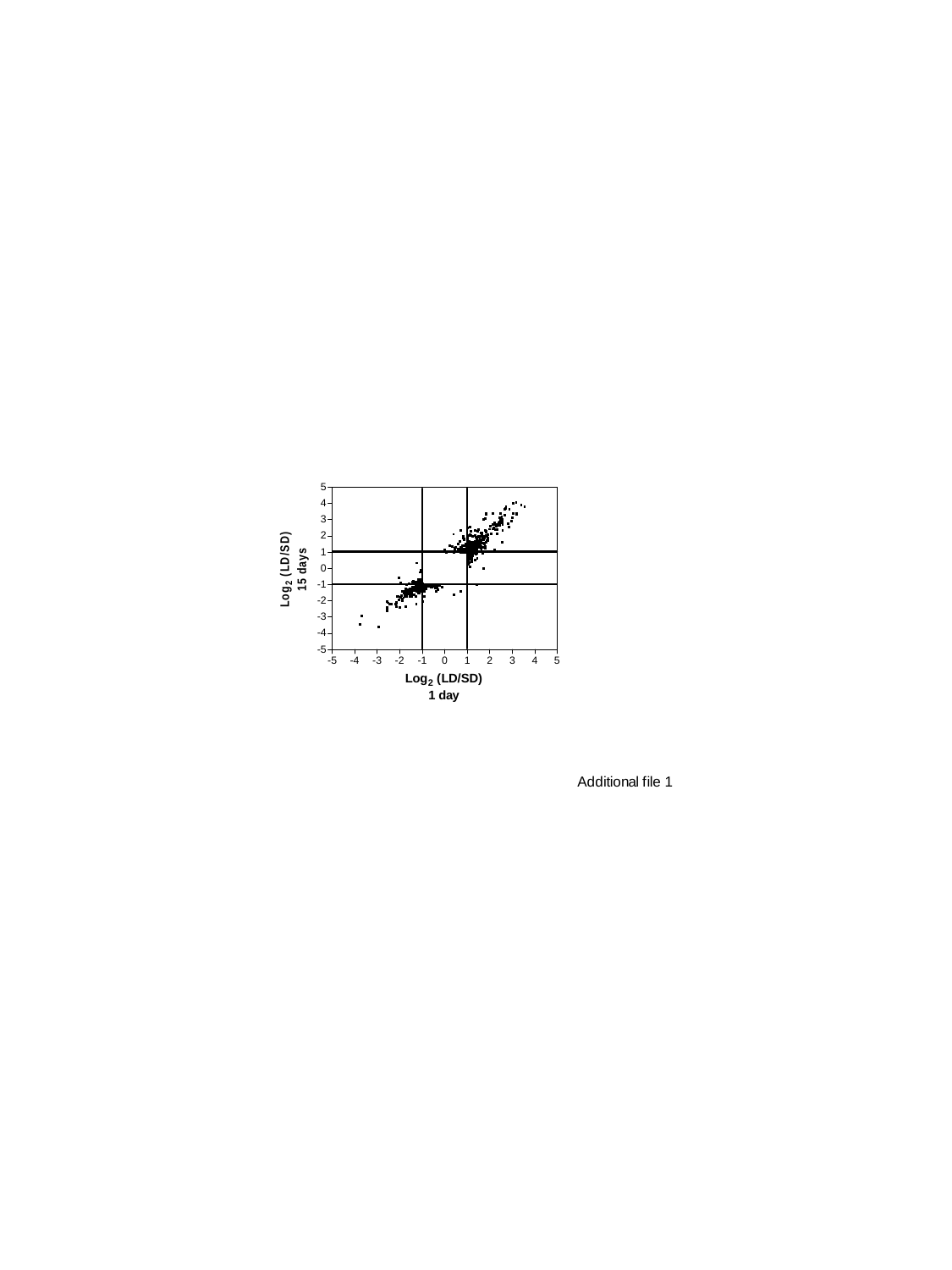

Supplement: Additional file 1 — Correlative analysis of the effect of 1 or 15 SD on gene expression in potato plants. XY graph comparing the expression ratios (log2LD/SD) of potato plants transferred from LD to SD conditions for 1 (x-axis) or 15 (y-axis) days. [file 1471-2164-10-60-S1.ppt]

## Slide 1
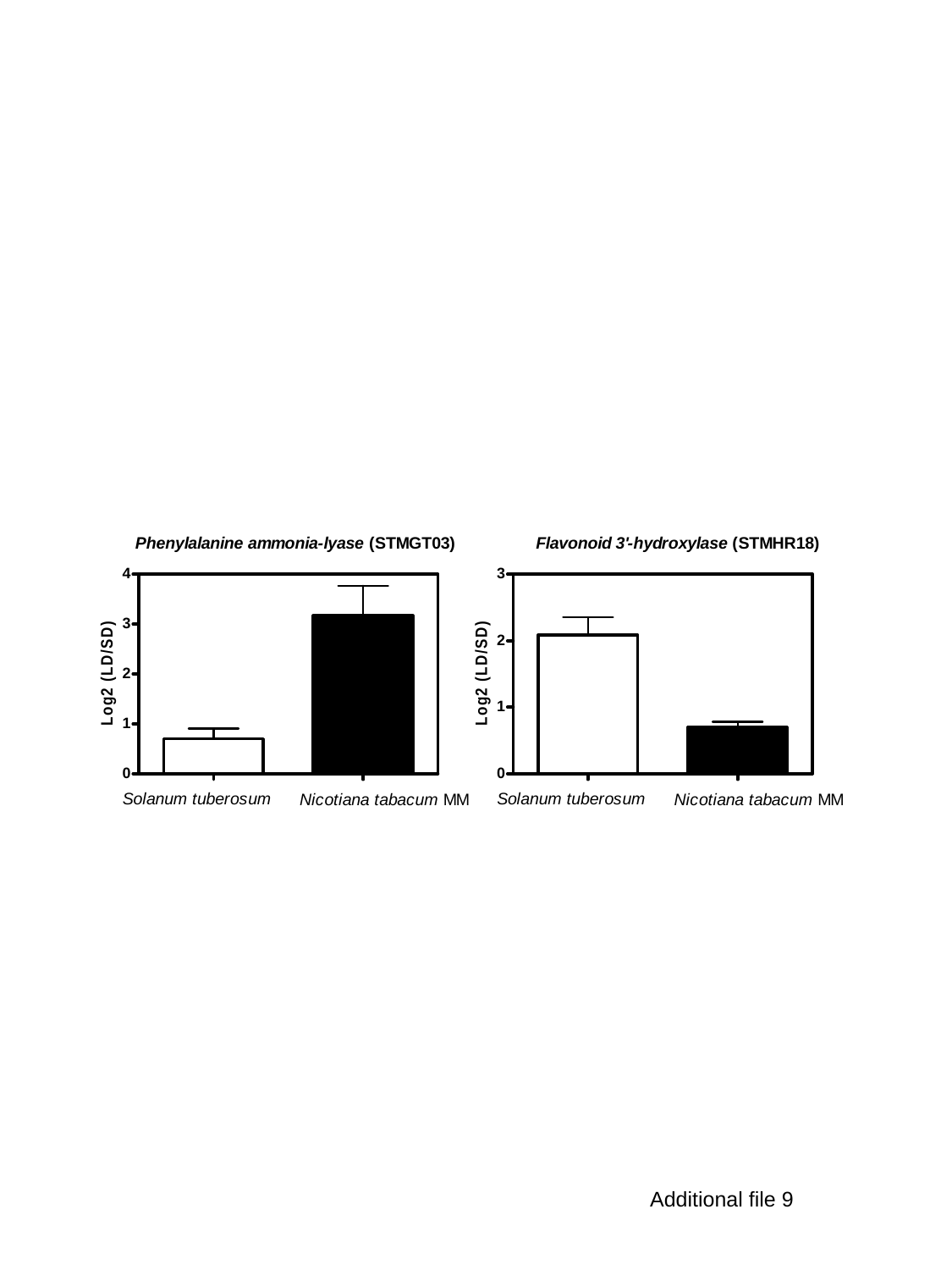

Additional file 9

Supplement: Additional file 9 — Contrasting response to photoperiod in potato and Nicotiana tabacum MM. Expression data corresponding to genes of the phenylpropanoid biosynthetic pathway that showed differential responses to photoperiod between potato and tobacco. [file 1471-2164-10-60-S9.ppt]
